# Epipericardial fat necrosis as a differential diagnosis of acute chest pain: a case report and algorithm proposal for diagnostic approach

**DOI:** 10.31744/einstein_journal/2023RC0183

**Published:** 2023-05-23

**Authors:** Tarso Augusto Duenhas Accorsi, Milena Ribeiro Paixão, Erick de Moraes Santos Piorino, Karine De Amicis, Karen Francine Köhler, José Leão de Souza

**Affiliations:** 1 Hospital Israelita Albert Einstein São Paulo SP Brazil Hospital Israelita Albert Einstein, São Paulo, SP, Brazil.

**Keywords:** Chest pain, Fat necrosis, Pericardium, Emergency medicine, Algorithms

## Abstract

Chest pain is a frequent, potentially life-threatening condition in the emergency department and requires immediate investigation and treatment. This case report highlights a rare differential diagnosis of pleuritic chest pain: epipericardial fat necrosis. A 29-year-old man presented with normal clinical evaluation, electrocardiography, point-of-care ultrasound, and unremarkable laboratory tests. The initial hypothesis was acute pleuritis. Chest radiography revealed peri-cardiac nonspecific findings, and computed tomography revealed epicardial fat necrosis. Despite the rarity of this condition, accurate diagnosis allows for better practices. An algorithm for a diagnostic approach is proposed.

## INTRODUCTION

Epipericardial fat necrosis (EFN) is a rare, benign cause of pleuritic chest pain in young patients that can mimic situations such as pericarditis, pulmonary embolism, and myocardial infarction.^( 1 )^ Although the first reported EFN case in the indexed literature was in 1957 and some reports focusing on tomography findings have been published since then, until now, there are no comprehensive guidelines for diagnosis suspicion and treatment. Herein, we report a case of a patient with acute pleuritic chest pain presented to the emergency department (ED).^( 2 , 3 )^ This report aimed to highlight the possible diagnosis of EFN in low-risk patients and to review the literature focusing on red flags for an accurate diagnostic approach. Moreover, an algorithm for a diagnostic approach is suggested.

## CASE REPORT

**Figure 1:**
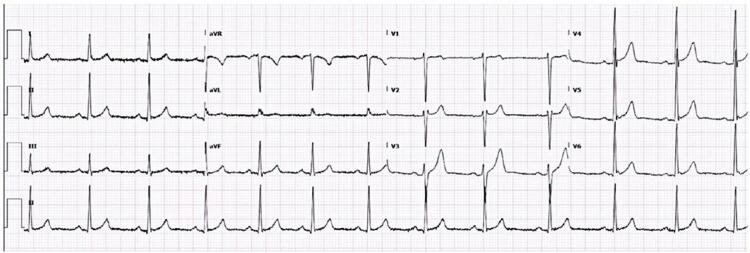
Electrocardiogram: early repolarization

**Figure 2:**
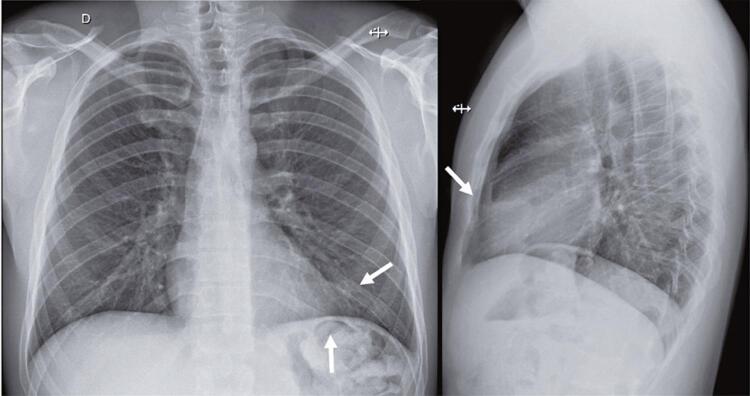
Chest radiograph: elevation of the left dome of the diaphragm and blurring of the cardiac silhouette in the posteroanterior view and opacity of the anterior base of the thorax in the lateral view (white arrows)

**Figure 3:**
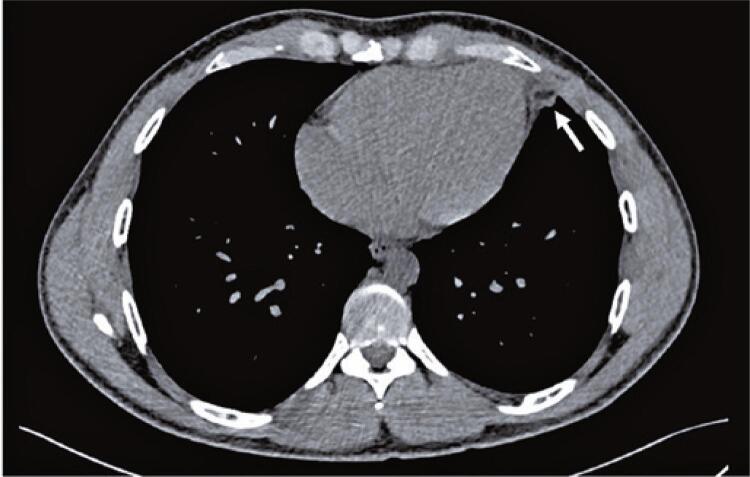
Computed tomography of the chest on axial view evidencing a rounded lesion (arrow) with fat attenuation and variable degrees of densification of the adipose planes

**Figure 4:**
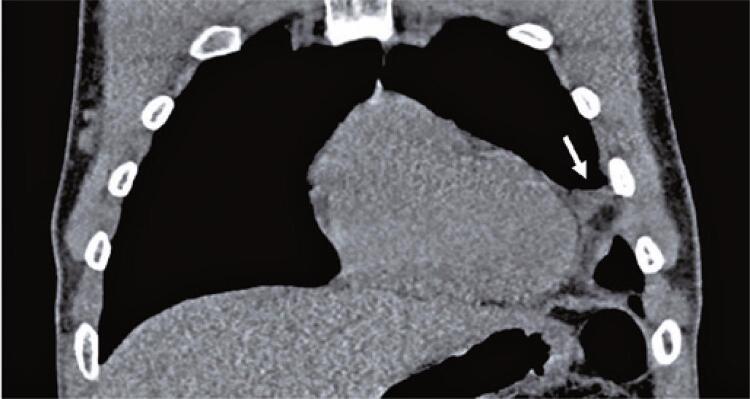
Computed tomography of the chest on coronal view evidencing a rounded lesion (arrow) with fat attenuation and variable degrees of densification of the adipose planes

A 29-year-old man presented to the ED with 6-day non-traumatic pleuritic chest pain. The pain was stabbing, intense, and severe in the dorsal decubitus position. On admission, the vital signs were as follows: blood pressure, 136/89 mmHg; heart rate, 92 beats per minute; respiratory rate, 15 breaths per minute; oxygen saturation, 98% in ambient air; and temperature, 36.6°C. Cardiovascular and pulmonary examinations showed no pericardial friction rubbing and no other remarkable findings. Electrocardiography revealed early repolarization ( Figure 1 ). Chest radiography revealed elevation of the left dome of the diaphragm, tenuous blurring of the cardiac silhouette, and opacity of the anterior base of the thorax ( Figure 2 ). Point-of-care ultrasonography showed no signs of pericarditis or cardiac effusion. The C-reactive protein was 7.3mg/L (normal range <5mg/L), and the hemogram, D-dimer, and troponin levels were within the normal range. He underwent chest computed tomography (CT) to diagnose EFN ( Figure 3 and 4 ). He was discharged with a mid-term anti-inflammatory strategy.

This study was approved by the Research Ethics of *Hospital israelita Albert Einstein* under CAAE: 52727421.2.0000.0071; # 5.131.433.

## DISCUSSION

Non-traumatic acute chest pain represents 8% of all ED encounters per year in the United States.^( 4 )^ Acute coronary syndrome is the leading life-threatening condition and reason for initial work-up.^( 5 )^ Regarding non-cardiovascular etiologies, musculoskeletal chest pain is the most prevalent etiology (44.7%), followed by psychiatric (4%), gastrointestinal tract (2.6%), and pulmonary disorders (2.2%). Nevertheless, approximately 46% of patients are discharged with a non-specific diagnosis of chest pain, which may include possible undiagnosed cardiovascular causes.^( 6 )^ Despite the appropriate tendency for early discharge, some conditions may require further investigation and specific treatment.^( 7 )^

Epipericardial fat necrosis usually presents as a new-onset pleuritic chest pain in young healthy patients.^( 8 , 9 )^ It is a benign, self-limiting condition; however, it may be associated with anxiety, multiple tests to investigate severe chest pain, and ED readmissions.^( 10 )^ Although its pathogenesis remains uncertain, the main hypotheses are ischemia resulting from spontaneous torsion of the vascular pedicle or capillary rupture due to an increase in intrathoracic pressure related to the Valsalva maneuver.^( 2 , 3 )^ Usually, EFN diagnosis is incidental after imaging tests are ordered based on other cardiac and pulmonary suspicions.^( 11 )^ There are case reports in the indexed literature and some reviews; however, to date, clinicians are unfamiliar with this condition. Diagnosis is almost always incidental, and previous studies have not suggested a comprehensive approach for suspected cases.^( 12 )^

The main manifestation of EFN is pleuritic pain, typically characterized by sharp and localized thoracic or shoulder pain and exacerbated by deep breathing, coughing, or chest movement.^( 13 )^ Typical physical examination findings include tachycardia, tachypnea, and even cardiac friction rub can be found.^( 8 )^ Potentially serious conditions may be related to these manifestations, such as pericarditis, pneumothorax, pulmonary embolism, acute coronary syndrome, and acute aortic syndrome.^( 14 , 15 )^

**Figure 5:**
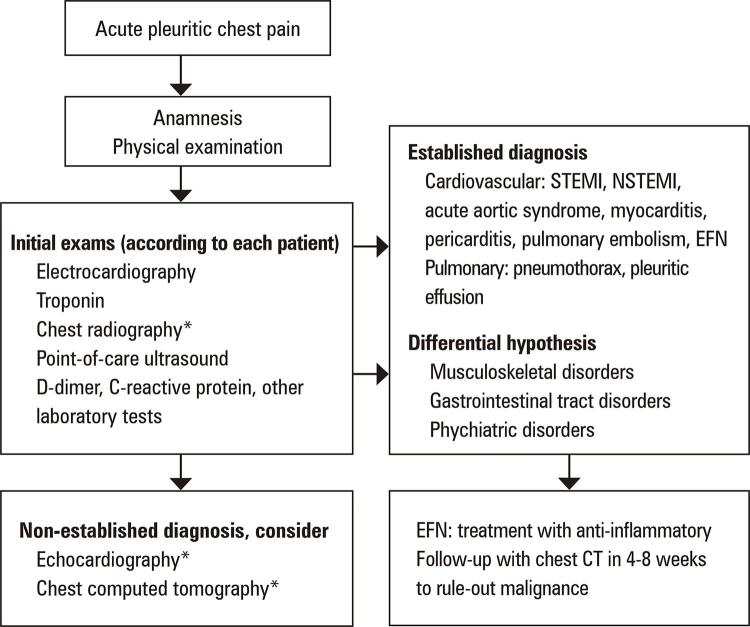
Proposed algorithm for acute pleuritic chest pain in the emergency department * Examiners should be aware of the EFN hypothesis. STEMI: ST-segment elevation myocardial infarction; NSTEMI: non (persistent) ST-segment elevation myocardial infarction; EFN: epipericardial fat necrosis; CT: computed tomography.

Epipericardial fat necrosis rarely manifests as electrocardiogram changes (eventually nonspecific repolarization findings).^( 12 )^ Blood samples may reveal mild increase in C-reactive protein, D-dimer, and white blood cell levels. Chest radiography rarely shows pericardial thickening, paracardiac opacity, pleural effusion, or atelectasis.^( 12 , 14 )^ Epipericardial fat necrosis was observed in 0.26% of the radiographs performed to investigate chest pain in the ED.^( 16 )^ There are very few current reports on point-of-care ultrasound and echocardiography in this setting. Some researchers have found hyperechoic nodules in epicardial fat surrounded by a hypoechoic halo and hyperechogenic adjacent fat tissue. The gold standard for EFN diagnosis is pericardial thickening and an encapsulated fatty lesion with dense strands on CT or magnetic resonance imaging.^( 17 - 19 )^ A retrospective review of 7,463 chest CT scans performed in the ED to investigate chest pain found 2.15% of EFN, with and without pleural effusion.^( 20 )^ Radiologists should be aware of this suspicion as EFN is frequently misdiagnosed even with these examinations.^( 21 )^ Differential diagnoses include primary fatty tumors (lipoma, liposarcoma, teratoma, and thymolipoma), diaphragmatic hernias, and mediastinitis.^( 18 )^ To clarify this hypothesis and manage EFN, an algorithm for a diagnostic approach is proposed ( Figure 5 ).

Treatment includes anti-inflammatory therapy and follow-up chest CT at 4-8 weeks to confirm healing and rule out tumors. The prognosis is good, and there have been no reports of chronic pain, recurrence, chronic pericarditis, or tamponade.^( 5 - 7 , 10 - 13 , 16 - 21 )^

## CONCLUSION

This case report describes the most common presentation of epipericardial fat necrosis in young healthy patients with pleuritic acute chest pain and negative emergency department work-up for more prevalent cardiovascular and pulmonary etiologies. Chest radiography revealed mild changes. The limited image resolution and lack of suspicion for this disorder did not lead to a diagnosis at the first emergency department visit. Chest tomography, performed to explore the differential diagnosis after readmission, revealed epipericardial fat necrosis. The patient’s condition improved with nonsteroidal anti-inflammatory therapy. This is an illustrative case to keep epipericardial fat necrosis diagnosis in mind.
